# Supplementary figures and images for: Misperception of the facial appearance that the opposite-sex desires
Source: PLoS One. 2024 Nov 14;19(11):e0310835. doi: 10.1371/journal.pone.0310835 (PMC11563419; doi:10.1371/journal.pone.0310835)

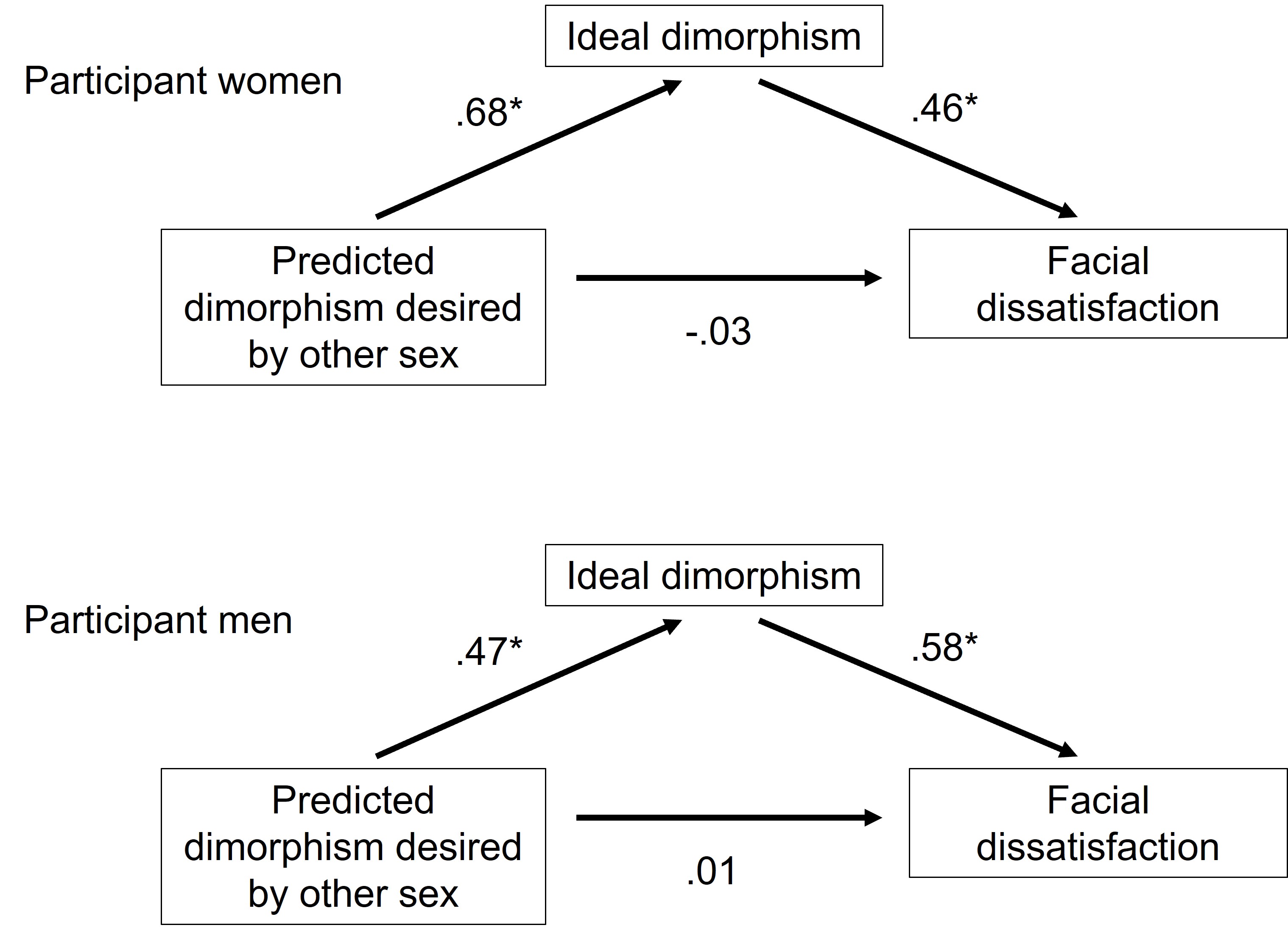

Supplement: S1 Fig — Linear regression models of the direct and indirect effects of facial dimorphism predicted to be desired by the opposite sex for participant women (upper) and men (lower). The flow chart shows the standardised independent direct effects (β values, * p < 0.05) of predicted dimorphism desired by the opposite sex on ideal dimorphism and facial dissatisfaction. (JPG) [file pone.0310835.s001.jpg]
