# Supplementary material for: Misperception of the facial appearance that the opposite-sex desires
Source: PLoS One. 2024 Nov 14;19(11):e0310835. doi: 10.1371/journal.pone.0310835 (PMC11563419; doi:10.1371/journal.pone.0310835)
Supplement: S1 File — (DOCX) [file pone.0310835.s002.docx]

Misperception of the facial appearance that the opposite-sex desires

David I. Perrett^1,*^, Iris J. Holzleitner^1,2^ and Xue Lei^1,3^

**Supporting information**

In the main text reported the relationship between misperception of opposite-sex desires and facial dissatisfaction. Here we report a similar but stronger relationship with ideal facial dimorphism.

For participant women, mixed ANCOVA was conducted with prediction of men’s desires for femininity in short- and long-term relationships as a repeated measure and ideal dimorphism as a covariate. This analysis revealed a main effect of ideal dimorphism (F_[1,69]_ = 59.64, p < .001, η_p_^2^ = .464). The more exaggerated the women’s prediction of facial femininity desired by men was, the greater the women’s ideal facial femininity. There was no main effect of relationship term (F_[1,69]_ = 0.005, p = .941, η_p_^2^ = .000) and no interaction between term and the ideal dimorphism (F_[1,69]_ = 0.83, p = .366, η_p_^2^ = .012).

For participant men, mixed ANOVA was conducted with prediction of women’s desires for male facial masculinity in short- and long-term relationships as a repeated measure and ideal dimorphism as a covariate. This analysis revealed a main effect of ideal dimorphism (F_[1,70]_ = 20.28, p < .001, η_p_^2^ = .225). The more exaggerated the men’s prediction of facial masculinity desired by women was, the greater the ideal facial masculinity. There was no main effect of relationship term (F_[1,70]_ = 0.78, p = .381, η_p_^2^ = .011) and no interaction between term and the ideal dimorphism (F_[1,70]_ = 2.60, p = .111, η_p_^2^ = .036).

This pair of analyses shows a stronger relationship between ideal dimorphism and misperception of opposite-sex desires (effect sizes for men and women .464 and .225, respectively) than that between dissatisfaction and misperception (effect size .081 and .073). Indeed, the larger effect size for ideal dimorphism than for facial dissatisfaction suggests that ideal dimorphism mediates the relation between misperception of opposite-sex desires and facial dissatisfaction.

To further investigate the relationships between prediction of opposite-sex desires, ideal dimorphism and dissatisfaction, a mediation analysis was performed. Since ANCOVA’s showed no differential effect of long- and short-term relationship contexts on ideal dimorphism or dissatisfaction the two relationship contexts were averaged together. For the mediation analysis the average prediction of dimorphism across short- and long-term relationships preferred by the opposite sex was the independent variable, ideal dimorphism was the mediator and facial dissatisfaction was the outcome variable (see S1 Fig).

For women, the relationship between women’s prediction of men’s desires for female facial femininity and ideal dimorphism was computed with linear regression. The overall model was significant (F[1,69] = 59.64, p <.001) with the unstandardised coefficient B = 0.752, SE = 0.097, p < .001). Regression analysis, with both opposite-sex desires and ideal dimorphism as predictors of dissatisfaction as the dependent variable, revealed a significant model overall (F[1,69] = 6.76, p = 002). In this model the mediator, ideal dimorphism, had a significant relation to dissatisfaction (B = 0.598, SE = 0.213, p = .007) but the independent variable, prediction of opposite-sex desires, was not significantly related to the facial dissatisfaction index (B = -0.041, SE = 0.235, p =.862 see S1 Fig, upper).

For men, the relationship between men’s prediction of women’s desires for male facial femininity and ideal dimorphism was computed with linear regression. The overall model was significant (F[1,69] = 20.277, p <.001; B = 0.556, SE = 0.124, p = < .001). Regression analysis, with prediction of opposite-sex desires as the independent variable and ideal dimorphism as the mediator and facial dissatisfaction as the dependent variable, revealed a significant model overall (F[1,69] = 17.27, p <.001). In this model the mediator, ideal dimorphism, had a significant relation to facial dissatisfaction (B = 0.572, SE = 0.110, p < .001) but the independent variable, prediction of opposite-sex desires, was not significantly related to facial dissatisfaction (B =.009, SE = 0.126, p =.945, see Fig S1, lower).

Using the coefficients from these regression analyses in mediation tests confirmed that ideal dimorphism significantly mediated the relationship between predictions of opposite-sex desires and facial dissatisfaction for both participant women (Sobel Test = 3.396, SE = 0.094, p < .001) and participant men (Sobel Test = 2.859, SE = 0.169, p = .004).

**SI Fig. Predictors of facial dissatisfaction.** Linear regression models of the direct and indirect effects of facial dimorphism predicted to be desired by the opposite sex for participant women (upper) and men (lower). The flow chart shows the standardised independent direct effects (β values, * p < 0.05) of predicted dimorphism desired by the opposite sex on ideal dimorphism and facial dissatisfaction.
