# Supplementary material for: Misperception of the facial appearance that the opposite-sex desires
Source: PLoS One. 2024 Nov 14;19(11):e0310835. doi: 10.1371/journal.pone.0310835 (PMC11563419; doi:10.1371/journal.pone.0310835)
Supplement: Attachment — Submitted filename: Response to Reviewers.docx [file pone.0310835.s003.docx]

PONE-D-24-22275

Misperception of the facial appearance that the opposite sex desires

Dear Johannes Hönekopp

We thank you and the 2 reviewers for the valuable comments. We have addressed each point in text below. Where changes have been made in the manuscript this is indicated in yellow, both in response to the comments and in the revised manuscript with track changes.

David Perrett

Dear Dr. Perrett,

Thank you for submitting your manuscript to PLOS ONE. After careful consideration, we feel that it has merit but does not fully meet PLOS ONE’s publication criteria as it currently stands. Therefore, we invite you to submit a revised version of the manuscript that addresses the points raised during the review process.

 = = = = = = = = = = = = = = = = = = = = = = = = = = = = = =

Both reviews, for which I am grateful, state that your paper makes an important contribution and emphasize that representativeness is an issue that needs addressing, which agrees with my own reading. Please can I ask you to carefully address all points raised, with particular emphasis on representativeness. In this context you might find Bovet, Tognetti & Pollet (2022) helpful.

**Response.** Bovet et al. (2022) draw attention to the limited size of samples used to create some prototypes. Holtzman (2018) creates a prototype of high psychopathy from a small subset of 33 males. Indeed the same set of male faces contributed to prototypes for 47 behavioural traits. Bovet et al. (2022) make valid criticisms of prototype limitations.

We used a large set of faces (n = 118) to construct the sex dimorphism vector. Furthermore, we tested the external validity of the sexual dimorphism vector. This was done by assigning each of the 118 faces a morphological masculinity score derived from the within-set sexual dimorphism vector [49]. Additionally, a second out-of-set sexual dimorphism vector was derived from an independent set of 40 male and 40 female faces [50]. This out-of-set dimorphism vector was used to define a second set of morphological masculinity scores for the 118 faces. The morphological masculinity scores from the within-set and the out-of-set sexual dimorphism vectors were correlated (N = 118, Pearson’s r = .666).

This is described in text: “Thus, each of the 118 faces contributed to the definition of the dimorphism vector along which base faces were manipulated. Importantly, we compared the sexual dimorphism from a second independent set of 40 male and 40 female faces [50]. The sexual dimorphism vectors from the within-set and the independent out-of-set faces were both used to assign a morphological masculinity score [49,50] for each of the 118 faces. The two sets of scores correlated (N = 118, Pearsons r = .666). This shows that the face sexual dimorphism shape vector has external validity.”

We acknowledge the limitations in text “An improvement to the generalisability of the study would be to use sexual dimorphism from more than one set of faces.”

I would also like you to address the following points:

- Please state clearly if you pre-registered your hypotheses and, if applicable, where the pre-registration can be found.

We now state that the hypotheses were not pre-registered. “The study hypotheses were not pre-registered.”

- Please justify your sample size (e.g., Lakens, 2022, Collabra).

The methods now include sample size considerations. “Lei and Perrett [12] reported misperception of body shape (body mass index) preference between men and women with an effect size ranging between d = 0.4 and d = 0.9. If we assume that misperception of facial shape preferences has a similar range in effect size we can estimate an appropriate sample size for difference between two independent means (samples). With a power of 80%, an alpha error of 0.05 and an effect size of d = 0.4, G-power suggests a sample size of 76 men and 76 women.”

- L76, “good quality”: please explain.

Quality here is defined in the specialised and limited sense of competition in the ‘mating market’ [25]. We have added: “Good quality from this limited perspective refers to individuals that are desired as a partner, and who can out-compete rivals in their own quest to secure a desirable partner. Multiple attributes contribute to mate quality (including resources, youth and physical attractiveness).”

We conclude the paragraph by noting: “Hence, facial masculinity in men can be considered as a sign of quality in the mating market”.

- L129, “participants’ estimate of their ideal”: Why is this an estimate? Reconsider wording.

The sentence has now been simplified and the term “estimate” deleted: “This index can be computed as the difference between the participant’s ideal facial dimorphism and the participant’s own facial dimorphism (ideal minus own dimorphism).”

- L138, “Long-term relationships come with direct benefits of parental investment”: Benefit for whom?

The recipient of direct benefits would be *the participant* expressing mate preferences. This is now explicitly stated: “Long-term relationships come with direct benefits (to the participant) of parental investment, resources and safety, whereas short-term relationships can have indirect genetic benefits (to the participant’s offspring) but only temporary resources [44].”

- L153, “we will”: reconsider tense.

“Will” is deleted: “To test this, we measure the male face shape that participant women desire and compare this to the face shape that men predict women will desire. Reciprocally, we measure the female face shape that participant men desire.”

- L203, “composite stimuli … more representative”: Of what? Reference needed.

Composite facial stimuli have a long history. Composites made by combining several facial images average out idiosyncratic or inconsistent features and maintain features that are common to a group. We now explain and cite a review of composite facial stimuli.

“Composite stimuli were employed because they are more representative (of the category of faces from which they were constructed) than the original faces [48]. Additionally, the composite images cannot be recognised as known individuals.”

“[48]. Perrett DI. Representations of facial expressions since Darwin. Evolutionary Human Sciences, 2022;4, e22 doi: 10.1017/ehs.2022.10”

- L209, “with the distance from the average female face to the average male face being equivalent to a +100% increase”: Please indicate SDs within female and male stimuli using this measure. (In other words, what is the effect size for the female/male difference in the stimuli?)

The following text has been added to answer the question about SD and effect size. “To provide more information on the shape transformation applied [49], shape dimorphism score varied within the original sample of 68 female faces SD = 36.5% and within the original sample of 50 male faces SD = 42.8%. The difference in facial shape dimorphism scores between the original sample of male and female faces had an effect size Cohen’s D = 0.72.”

- L213 and L223 seem to contradict each other.

Line 213 refers to the ‘base’ faces which were subsequently transformed in sexually dimorphic shape (as described in line 223) to create a range in appearance that the participants chose.

This has been clarified in text: “The study used 3D stimuli. The stimuli were two male and two female ‘base’ faces. Each of the male base faces was a composite or average of the shape, colour and texture of three male faces. Likewise, each of the female base faces was a composite of shape, colour and texture of three female faces.

And

“The two male base faces were each feminised and masculinised in shape within a range from −100% to +200%, and the two female base faces were each masculinised and feminised in shape within a range from −100% to +300% sexual dimorphism.”

- L232, “presented with a series of male and female face”: How many? If there was more than one stimulus for each DV, please provide some information on reliability/internal consistency.

There were 2 male face stimuli, or 2 female face stimuli used for each facial judgment. Measures of consistency have now been added. “Participants’ selection of sexual dimorphism of the two faces was correlated for each judgment. For the 72 men, judgments for the 2 example faces correlated for own facial masculinity (r = .67), ideal facial masculinity (r = .82), prediction of male facial masculinity preferred by women for short- (r = .72) and long-term relationships (r = .69) and preference for female facial femininity for short- (r = .59) and long-term (r = .66) relationships. Likewise for the 72 women, judgments for the 2 example faces correlated for own facial femininity (r = .72), ideal facial femininity (r = .60), prediction of female facial femininity preferred by men for short- (r = .70) and long-term relationships (r = .63) and preference for male facial masculinity for short- (r = .69) and long-term (r = .72) relationships. Given the consistent and high correlation, the values for the 2 example faces for each judgment were averaged together and the average values entered as dependent variables for analysis in SPSS.”

- L336: I don’t see how perceived level of own facial masculinity is a sensible predictor in your regression, given that it was already used to compute your criterion.

**Response**. We acknowledge the editor’s point. Using own dimorphism to predict dissatisfaction (own - ideal dimorphism) may reflect the impact of ideal dimorphism. Therefore, we now use a different statistical approach to demonstrate the relation between the misperception of opposite-sex desires and the facial dissatisfaction index. Using ANCOVA we show that the dissatisfaction index (as the sole covariate) is related to the of misperception of opposite-sex desires.

Additionally, in supplementary information we include ANCOVA results that show ideal dimorphism (as the sole covariate) is strongly related to the misperception of opposite-sex desires. Indeed, the larger effect size for ideal dimorphism suggests that it is mediating the relation between misperception of opposite-sex desires and the dissatisfaction index facial. Mediation analysis included in the supplementary information confirmed the mediation role of ideal dimorphism.

**Main body text. Results**

“For participant women, ANCOVA (analysis of covariance) with prediction of men’s desires of female facial femininity for short- and long-term as a repeated measure and facial dissatisfaction as a covariate showed a main effect of facial dissatisfaction (F_[1,69]_ = 6.12, p = .016, η_p_^2^ = .081). The greater the facial femininity women predicted men desired, the higher the women’s facial dissatisfaction was. There was no main effect of relationship term (F_[1,69]_ = 2.57, p = .113, η_p_^2^ = .036) and no interaction between relationship term and the facial dissatisfaction index (F_[1,69]_ = 2.29, p = .135, η_p_^2^ = .032).

For participant men ANCOVA was conducted with prediction of women’s desires of male facial masculinity for short- and long-term relationships as a repeated measure and facial dissatisfaction as a covariate. This analysis showed a main effect of facial dissatisfaction (F_[1,69]_ = 5.41, p < .023, η_p_^2^ = .073). The greater the facial masculinity men predicted women desired, the higher the men’s facial dissatisfaction score was. There was a main effect of relationship term (F_[1,69]_ = 6.23, p = .015, η_p_^2^ = .083) reflecting men’s prediction that women preferred higher levels of facial masculinity for short- rather than long-term relationships. There was no interaction between relationship term and the facial dissatisfaction index (F_[1,69]_ = 0.08, p = .775, η_p_^2^ = .001).

Equivalent ANCOVAs with ideal dimorphism as a covariate show that it is strongly related to the misperception of opposite-sex desires (see Supplementary Information). Mediation analysis included in the supplementary information confirmed the mediation role of ideal dimorphism in the relation between misperception and dissatisfaction.”

**Main body text. Discussion.**

“Analysis in supplementary information suggested that the relationship between misperception of opposite-sex desires and facial dissatisfaction was mediated by ideal dimorphism. Misperception of desires was directly associated with exaggerated ideals and exaggerated ideals were in turn directly associated with a higher facial dissatisfaction score. The direct pathway between misperception and dissatisfaction was non-significant when both ideals and misperception were used as predictors of dissatisfaction.”

- L499, “involved White models and predominantly White UK participants”: How does this agree with your “White ethnicity” pre-screening criterion (L191)?

Thank you for picking up this error. All participants gave current country of residence as UK. The text has been changed to read: “Our study involved White models and White participants currently resident in the UK.”

= = = = = = = = = = = = = = = = = = = = = = = = = = = = = =

Please submit your revised manuscript by Aug 24 2024 11:59PM. If you will need more time than this to complete your revisions, please reply to this message or contact the journal office at [plosone@plos.org](mailto:plosone@plos.org). When you're ready to submit your revision, log on to <https://www.editorialmanager.com/pone/> and select the 'Submissions Needing Revision' folder to locate your manuscript file.

Please include the following items when submitting your revised manuscript:

- A rebuttal letter that responds to each point raised by the academic editor and reviewer(s). You should upload this letter as a separate file labeled 'Response to Reviewers'.
- A marked-up copy of your manuscript that highlights changes made to the original version. You should upload this as a separate file labeled 'Revised Manuscript with Track Changes'.
- An unmarked version of your revised paper without tracked changes. You should upload this as a separate file labeled 'Manuscript'.

Kind regards,

Johannes Hönekopp
Academic Editor
PLOS ONE

Journal Requirements:

1. When submitting your revision, we need you to address these additional requirements.

Please ensure that your manuscript meets PLOS ONE's style requirements, including those for file naming. The PLOS ONE style templates can be found at 
[https://journals.plos.org/plosone/s/file?id = wjVg/PLOSOne_formatting_sample_main_body.pdf](https://journals.plos.org/plosone/s/file?id=wjVg/PLOSOne_formatting_sample_main_body.pdf) and 
[https://journals.plos.org/plosone/s/file?id = ba62/PLOSOne_formatting_sample_title_authors_affiliations.pdf](https://journals.plos.org/plosone/s/file?id=ba62/PLOSOne_formatting_sample_title_authors_affiliations.pdf)

Formatting has been standardised following PLoS One recommendations

2. Thank you for uploading your study's underlying data set. Unfortunately, the repository you have noted in your Data Availability statement does not qualify as an acceptable data repository according to PLOS's standards.

At this time, please upload the minimal data set necessary to replicate your study's findings to a stable, public repository (such as figshare or Dryad) and provide us with the relevant URLs, DOIs, or accession numbers that may be used to access these data. For a list of recommended repositories and additional information on PLOS standards for data deposition, please see <https://journals.plos.org/plosone/s/recommended-repositories>.

We have uploaded data to figshare. We also maintain the data deposit at OSF.

“The data that support the findings of this study are openly available on the Open Science Framework at [https://osf.io/3rkxz/?view_only = 46a7dd9adae048218c328ab9a912b331](https://osf.io/3rkxz/?view_only=46a7dd9adae048218c328ab9a912b331) . The data are also available at [https://figshare.com/articles/dataset/Misperception_of_the_desirability_of_sexual_dimorphism/26424307?file = 48071893](https://figshare.com/articles/dataset/Misperception_of_the_desirability_of_sexual_dimorphism/26424307?file=48071893)”

3. We note that Figures 1, 3 and 5 includes an image of a participant in the study. 
As per the PLOS ONE policy (<http://journals.plos.org/plosone/s/submission-guidelines#loc-human-subjects-research>) on papers that include identifying, or potentially identifying, information, the individual(s) or parent(s)/guardian(s) must be informed of the terms of the PLOS open-access (CC-BY) license and provide specific permission for publication of these details under the terms of this license. Please download the Consent Form for Publication in a PLOS Journal ([http://journals.plos.org/plosone/s/file?id = 8ce6/plos-consent-form-english.pdf](http://journals.plos.org/plosone/s/file?id=8ce6/plos-consent-form-english.pdf)). The signed consent form should not be submitted with the manuscript, but should be securely filed in the individual's case notes. Please amend the methods section and ethics statement of the manuscript to explicitly state that the patient/participant has provided consent for publication: “The individual in this manuscript has given written informed consent (as outlined in PLOS consent form) to publish these case details”.

If you are unable to obtain consent from the subject of the photograph, you will need to remove the figure and any other textual identifying information or case descriptions for this individual.

**Response.** We note that the illustrations of facial images are not of real individuals. In Figure 1 the images contain the shape, colour and texture of 50 male faces averaged together, and 68 females faces averaged together. No individual can be recognised from the images.

We note that such average or composite facial images have been published in PlosOne previously. Three examples of PLoS One are listed below:

Little AC, et al. (2008). Symmetry is related to sexual dimorphism in faces: data across culture and species. *PLoS One*, *3*(5), e2106. Figure 3. High and low symmetry composite faces for macaques, Hadza, and Europeans. <https://doi.org/10.1371/journal.pone.0002106.g003>

Scott IM, et al. (2010). Does masculinity matter? The contribution of masculine face shape to male attractiveness in humans. *PLoS One*, *5*(10), e13585. Figure 1. Masculinity, measured, rated and morphed: examples of dimensions. To preserve anonymity of participants, these faces are composites rather than real individuals.…. <https://doi.org/10.1371/journal.pone.0013585.g001>

Sanchez-Pages S, et al. (2014). Facial masculinity: How the choice of measurement method enables to detect its influence on behaviour. *PLoS One*, *9*(11), e112157. Figure 1. Employed landmarks. A) An average face generated with the complete female population (n  =  74). <https://doi.org/10.1371/journal.pone.0112157.g001>

The legend now reads “**Fig 1: Transformation of sexually dimorphic face shape.** Upper: the average male face transformed by −100% (feminised), 0% (neutral), +100% (masculinised) and +200% (masculinised) sexual dimorphism. Lower: the average female face transformed by −100% (masculinised), 0% (neutral), +100% (feminised) and +200% (feminised) sexual dimorphism. To preserve anonymity of participants, these images are averages of the shape, colour and texture of 50 male faces and 68 female faces, respectively, rather than of real individuals.”

Fig 3 and 5 have now also been remade using the average male and average female faces. There legends have been amended to assure that no individuals are recognizable.

**“Fig 3. Male facial masculinity chosen**. Men’s prediction of women’s preferences (left) and women’s masculinity preference (right) for short-term and long-term relationship contexts. Numbers in white display the mean amount of sexual dimorphism transform applied. The facial images are averages of the shape, colour and texture of 50 male faces and 68 female faces, respectively, rather than of real individuals.”

And

**“Fig 5. Female facial femininity chosen.** (Left) Women’s prediction of men’s preferences for short-term and long-term relationship contexts. (Right) Men’s femininity preference for short-term and long-term relationship contexts. The facial images are averages of the shape, colour and texture of 50 male faces and 68 female faces, respectively, rather than of real individuals.”

**Reviewer #1:**

(1) The manuscript presents sound data with clear findings. Hypothesis set are derived from literature and unsurprisingly met in the findings. The stimuli is appropriate, though I question whether it is useful to include both front facing and three-quarter facing view within the same stimulus. Typically the face is viewed from the front and whilst body fat is easiest to see from three quarter view, for facial attractiveness, I believe, the front view would be sufficient. Please could further justification and discussion be added to address this concern.

The discussion now includes text acknowledging the potential benefit and limitation of using face and profile.

“Most research on facial appearance has been performed with frontal images of the face. The profile view of the head provides additional information about the neck, chin and cheek structure which may be important for attributions. Indeed, the profile pose of male faces decreases attractiveness and increases apparent dominance [51]. By including both face and half-profile views our results may have greater generality than studies using a single view. Future work testing the effects of the face, or the profile view alone could confirm the viewpoint specificity or generality of masculinity (mis)perceptions.”

(2) Furthermore, the stimulus is presenting Caucasian white male/female. It needs to be acknowledged that even within Caucasians, the facial features vary depending on which exact country a person originates from. Whist UK residency was an inclusion criteria, this cohort might include Scandinavian, Eastern European etc. participants whose facial features are very different to typical British facial features. Furthermore, how was it assured that the stimuli was presenting typical British facial features. Further discussion and justification required.

We acknowledge that the stimuli and participants include diversity within European populations. We restricted current residency to the UK, but future studies could explore population differences within and outside Europe. These limitations are included discussion.

“Our study involved White models (from diverse populations within Europe) and White participants currently resident in the UK. Future studies could explore population differences within and outside Europe extending to a range of ethnic groups, as body dissatisfaction shows consistent (but relatively small, d = 0.3) differences between White and Black women [64].”

(3) Data is analysed appropriately using a set of ANOVAs. It is though not clear why the participants' own relationship status is not included in the analysis. I believe this likely to have a contributing effect. Please discuss.

We did not collect information on participants’ partnership status and so were unable to include this factor in analysis. We now acknowledge the potential effect of partnership status as a limitation. “Partner’s status has been found to affect facial attractiveness judgments [65, 66] so could be included as an additional factor in future studies.”

[65] Lindová J, Little AC, Havlíček J, Roberts SC, Rubešová A, Flegr J. Effect of partnership status on preferences for facial self-resemblance. Frontiers in Psychology. 2016;7:869.

[66] Little AC, Jones BC, Penton-Voak IS, Burt DM, Perrett DI. Partnership status and the temporal context of relationships influence human female preferences for sexual dimorphism in male face shape. Proc R Soc Lond B Biol Sci. 2002;269(1496):1095-1100.

(4) My main concern is though 'so what'. Why is this important. Please could the authors add contemplation of why the current findings are important and how the line of research benefits the literature and general public knowledge. Clear indications of future research need to be equally identified.

We thank the reviewer for the chance to stress the novelty and potential utility of our results. We have added the following text to the conclusion.

“Research on appearance dissatisfaction has focused on body shape and composition. While there are cues to body weight and proportion of body fat evident in face shape [49], our research indicates that a common appearance concern reflects sex-typicality in facial appearance. Indeed, distorted ideals of body weight and muscularity may reflect underlying concerns about sex-typicality. Appearance dissatisfaction is an important issue as it contributes to mental and physical health. Our findings, therefore benefit the scientific literature and public knowledge by providing insight to the nature of harmful appearance ideals. Future research exploring strategies into moderate aberrant notions of (and attention to) sex-typicality in body or face shape may have benefits to self-esteem and health.

**Reviewer #2:** This was a well written manuscript with straightforward findings: both heterosexual men and women believe that the opposite sex prefers higher level of sexual dimorphism.

I would like to see a bit more discussion of the following points:

1. The scale for male and female stimuli was different, because in the case of male stimuli the highest exaggeration resulted in artifacts. But participants would generally adapt to the range of the scale and use it accordingly. If this is not accounted for, the results are a bit strange. First, there is about 100% difference between the responses for male and female faces. Second, this is also the case for self-assessment of facial shapes (37.2% for men vs. 124% for women). These results either simply reflect the very different ranges of the scales or strong bias in the case of female faces (or alternatively, very inaccurate perceptions).

**Response.** Yes, the average level for sexual dimorphism chosen for female faces was much higher than that for males. This may indeed reflect the different scales as is acknowledged in the discussion.

“Another explanation could be the use of ranges that were asymmetrical (for male faces, this was −100% to +200% and for female faces −100% to +300%). This might have briefly shifted participant’s perception of what is a “normal” level of sexual dimorphism and hence might have artificially inflated preferred levels of dimorphism (e.g., [63] particularly for female faces).”

The average level of dimorphism chosen is less important than the variation of dimorphism chosen in accordance with the beliefs of the participant. Women who believed that men prefer very feminine female faces were more likely to be dissatisfied with their own face shape.

2. Participants are making decisions about their own face shape. But how can we know that they are accurate at all?

We now acknowledge this subjectivity of self-report of face shape in discussion.

“We cannot assume complete accuracy in participant’s judgments about their own faces. Research using 3D scans of participants’ faces could define objective levels of sexual dimorphism in face shape. Nonetheless, subjective self-assessment is important as viewing attractive or unattractive others changes self-esteem and modifies face and body ideals [67,68].”

[67] Little AC, Mannion H. Viewing attractive or unattractive same-sex individuals changes self-rated attractiveness and face preferences in women. Animal Behaviour 2006;72(5), 981-987. doi: 10.1016/j.anbehav.2006.01.026

[68] Stephen ID, Perera AT. Judging the difference between attractiveness and health: does exposure to model images influence the judgments made by men and women? PloS one. 2014;20;9(1):e86302. doi: 10.1371/journal.pone.0086302

3. This is related to point 2 and the regression analyses. Relying on difference scores is tricky. It would be nice to see some raw data/ means before the differences are computed. A participant with 10% "self" shape and 50% "ideal" is different from one with 10% and 70% and possibly one with 30% and 70%.

This point is also reflected in the Editor’s question about regression (see above). Analysis with ideal dimorphism is clearer (less tricky) since it is not a difference score. Supplementary information shows that misperception is associated with ideal dimorphism. Analysis indicates that ideal dimorphism mediates the relation between misperception of opposite-sex desires and dissatisfaction.

We have made available all the data (inc. own and ideal dimorphism) in Figshare and Open Science Framework repositories. These allow interested parties to view the raw data and inspect difference scores.

“The data that support the findings of this study are openly available on the Open Science Framework at <https://osf.io/3rkxz/?view_only=46a7dd9adae048218c328ab9a912b331>. The data a also available at [https://figshare.com/articles/dataset/Misperception_of_the_desirability_of_sexual_dimorphism/26424307?file = 48071893](https://figshare.com/articles/dataset/Misperception_of_the_desirability_of_sexual_dimorphism/26424307?file=48071893)

Graphs of own (X axis) vs ideal dimorphism (Y axis) show that the two variables are positively correlated but have no obvious pattern beyond this. These graphs are included below for the reviewer. Such graphs show raw data before difference scores for facial dissatisfaction are computed. For most individuals, ideal dimorphism is greater than own dimorphism (i.e. data points lie above the diagonal in the graphs). The graphs are not included as they can be derived from uploaded files.

4. One conceptual point is that the main finding is based on the difference between predictions and preferences. But in principle, we don't know what population participants are having in mind when making their predictions. Another way to put it is that one needs to assume that samples are representative of the population of interest. At the very least, this needs to be discussed under limitations.

We now include the following text in limitations: “Choices were made within the context of specific instructions (e.g. “alter the male face to make it most attractive to heterosexual women for a short-term (sexual) relationship”). The instruction does not specify the population of women for whom the participant is to predict preference. Future research could target different populations differentiating between peer group and a younger or more attractive group.”
